# Anticoagulation in atrial fibrillation with factor X deficiency—A management dilemma

**DOI:** 10.1002/ccr3.1731

**Published:** 2018-07-22

**Authors:** Jian Liang Tan, Kah Poh Loh, Danielle Fortuna, Arezoo Ghaneie

**Affiliations:** ^1^ Department of Internal Medicine Crozer‐Chester Medical Center Upland Pennsylvania; ^2^ Division of Hematology and Oncology Strong Memorial Hospital/University of Rochester Medical Center Rochester New York; ^3^ Department of Pathology and Laboratory Medicine Perelman School of Medicine University of Pennsylvania Philadelphia Pennsylvania; ^4^ Associates in Hematology and Oncology, P.C. Crozer Regional Cancer Center Upland Pennsylvania

**Keywords:** anticoagulation, apixaban, atrial fibrillation, factor X deficiency

## Abstract

Factor X (FX) deficiency is a rare bleeding disorder. There is currently no clear guideline or recommendation for the appropriate selection of anticoagulation and management of patients with FX deficiency who require anticoagulation. We shared our experience in managing such patient, and we further discussed other possible treatment options.

## 1 INTRODUCTION

Factor X deficiency (FX) is a rare (inherited or acquired) blood coagulation disorder, which affects approximately one in 1 000 000 people. The most common cause of acquired FX deficiency is usually in association with amyloidosis. Patients with acquired FX deficiency have variable bleeding characteristics as patients with critically low FX level may have mild bleeding episode and vice versa.^1^, ^2^ Hence, FX levels do not often correlate well with the clinical bleeding risk.^2^ In this case report, we describe a patient with acquired FX deficiency who needs anticoagulation for paroxysmal atrial fibrillation (PAF). Patients with concurrent FX deficiency and atrial fibrillation (AF) requiring anticoagulation present several challenges and dilemmas due to concern for bleeding. To the authors' knowledge, no cases have been reported in the literature. There is also very limited guidance on the management of such rare clinical entity.

## 2 CASE PRESENTATION

A 64‐year‐old Caucasian woman with a history of acquired FX deficiency from Amyloid light‐chain (AL) amyloidosis, PAF, metastatic lung adenocarcinoma on erlotinib, hypertrophic cardiomyopathy (HCM), hypertension, and type 2 diabetes mellitus was seen in the hematology clinic for routine follow‐up.

**Figure 1:**
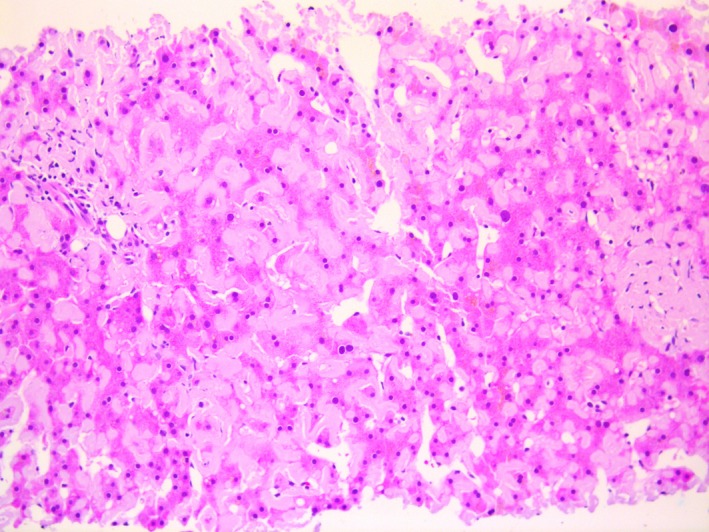
Liver biopsy shows diffuse deposition of eosinophilic, extracellular, amorphous material throughout the hepatic sinusoids consistent with amyloidosis involving the liver. A portal tract can be seen at the far left. (hematoxylin and eosin, 200× magnification)

**Figure 2:**
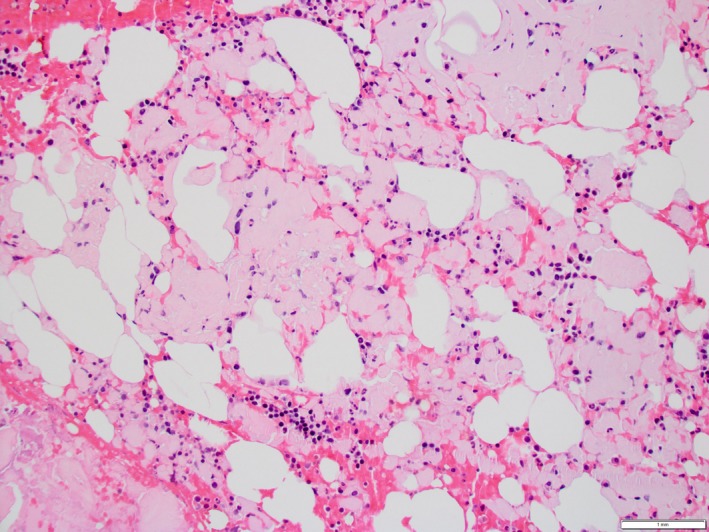
Bone marrow core biopsy specimen shows extensive acellular eosinophilic (interstitial amyloid) deposition consistent with AL amyloidosis. (hematoxylin and eosin, 200× magnification)

In 2013, she presented to the hematology clinic for self‐limiting epistaxis and easy bruising. Initial outpatient laboratory tests were significant for abnormal coagulation profile (prothrombin time (PT) of 12.4 seconds, INR of 1.2, and activated partial thromboplastin time (aPTT) of 38 seconds), elevated alkaline phosphatase (160 U/L), and creatinine (1.16 mg/dL) levels. Further investigations revealed proteinuria (882 mg/24 h), and abdominal ultrasound demonstrated hepatomegaly with the right lobe measuring 21 cm in craniocaudal dimension. Serum and urine electrophoreses were remarkable for the presence of a paraprotein (immunoglobulin G‐lambda). Abdominal fat pad biopsy was unrevealing, and liver biopsy (Figure 1) showed extensive amyloid deposition. Immunohistochemical staining of the liver tissue revealed the presence of lambda light chain. A bone marrow core biopsy demonstrated extensive amyloid deposition (Figure 2), and it was positive for Congo red staining with classical apple‐green birefringence under polarized light. Fluorescence in situ hybridization assay was positive for lambda light chains. Further work‐up of the abnormal coagulation study revealed a decreased FX coagulation activity of 45% (reference 70%‐150%). Screening for inhibitor to FX was negative. Hence, a diagnosis of acquired FX deficiency secondary to AL amyloidosis was made. In view of her recurrent lung adenocarcinoma, she was deemed a poor candidate for autologous stem cell transplantation and she was started on melphalan and dexamethasone chemotherapy.

In 2015, she developed an episode of symptomatic PAF. Her coagulation profile showed PT of 16.3 seconds, aPTT of 33 seconds, and FX activity of 34%. As she has HCM, her CHA_2_DS_2_‐VASc score was 4 with a significant risk for thromboembolism. Hence, she was started on apixaban 5 mg twice daily with close outpatient follow‐up.

Three months later, she was found to have progression of her lung adenocarcinoma as she sustained a left pathologic subtrochanteric fracture requiring an urgent surgical intervention. In view of the need for her to undergo an open reduction and internal fixation of the left hip, her apixaban was temporarily withheld for close to 30 hours prior to the surgery. Preoperatively, her laboratory values were as follows: hemoglobin of 9.7 g/dL, PT of 20.3 seconds, and aPTT of 36 seconds. She had an uneventful open reduction and internal fixation of her left hip requiring one unit of packed red blood cell transfusion. Postoperatively, her laboratory values were as follows: hemoglobin of 8.1‐8.4 g/dL, PT of 20.9 seconds, aPTT of 33 seconds, mixing studies showed correction (indicated underlying factor deficiency), fibrinogen level of 466 mg/dL, and FX coagulation activity of 37%. She was started on enoxaparin 30 mg subcutaneously every 12 hours for deep vein thrombosis prophylaxis. Two weeks later, she was restarted on her usual dose of apixaban for her paroxysmal AF. Her recovery was uneventful. A summary of her laboratory data is shown in Table 1.

**Table 1:** Laboratory data

Variable	Reference range^a^	Initial presentation in 2013	Prior to the initiation of apixaban in 2015	Three months after the initiation of apixaban (Preoperatively)	Apixaban was held for more than 30 h (Postoperatively)
Prothrombin time (s)	11.8‐14.7	12.4	16.3	20.3	20.9
INR	0.9‐1.1	1.2	1.3	1.8	1.9
Activated partial thromboplastin time (s)	22‐37	38	33	36	33
Factor X coagulation activity (%)	70‐150	45	34	N/A	37

N/A, not available.

a Reference values are affected by many variables, including the patient population and laboratory methods used.

## 3 OUTCOME AND FOLLOW‐UP

Our patient has been on apixaban 5 mg twice a day for the past one and a half years without any signs of overt bleeding or thromboembolic event. Nonetheless, the bleeding risk in a patient with amyloidosis‐related FX deficiency is unpredictable.^1^, ^2^ An implantable loop recording was performed, which did not detect any recurrence of AF. Apixaban was eventually discontinued.

## 4 DISCUSSION

AL amyloidosis is a systemic disorder due to underlying plasma cell dyscrasia, causing abnormal tissue deposition of misfolded immunoglobulin light‐chain proteins, which can result in progressive organ damage and death.^3^, ^4^ AL amyloidosis is commonly associated with coagulation abnormality; FX deficiency is the most common subtype occurring in 8.7% to 14% of patients with AL amyloidosis.^5^ FX is a crucial component of thrombin formation in the coagulation cascade. FX binds to amyloid fibrils and hence shortened its half‐life in the plasma.^6^ Other reported cases of acquired FX deficiency include liver disease, vitamin K deficiency, multiple myeloma, solid tumors, hematologic malignancy, bacterial/viral infection, drug‐induced (sodium valproate), and acquired inhibitors to FX.^2^

Atrial fibrillation is one of the most common atrial arrhythmias and remains as one of the leading preventable causes of stroke in the world.^7^ Patients with AF have a fivefold increased risk of stroke, and this risk increases with age.^8^ Anticoagulation is associated with long‐term risk reduction in stroke in AF. The American College of Cardiology/American Heart Association guideline issued a class I recommendation that all patients with AF should be assessed for risk of thromboembolism using the CHA_2_DS_2_‐VASc (congestive heart failure, hypertension, age ≥75 years, diabetes mellitus, prior stroke, transient ischemic attack, or thromboembolism, vascular disease, age 65‐74 years, sex category [female]) score. An oral anticoagulant is highly recommended for a score of ≥2 irrespective of the types of AF pattern (paroxysmal, persistent, or permanent).^8^ In our patient, she had PAF and HCM (corresponding to an overall 3.75% per year risk of stroke), and her CHA_2_DS_2_‐VASc score was 4 (corresponded to a 4% per year risk of stroke).^8^, ^9^ Therefore, she was started on an oral anticoagulant agent.

The following discussion focuses on the use of direct FXa inhibitor and other possible treatment options in a patient with acquired FX deficiency and AF. Due to rarity of acquired FX deficiency, there is no clear guideline for the management of AF in patients with FX deficiency. If oral anticoagulants were to be prescribed, the risks and benefits must be considered carefully. As reported by Greipp and colleagues, a patient with mild FX deficiency (FX level 40 IU/dL) had a life‐threatening hemorrhagic event.^1^ Our patient had a FX activity in the range of 34%‐45% and had not experienced any severe bleeding events perioperatively and throughout her clinical course despite being on anticoagulation.

As there is presently insufficient evidence regarding the appropriate choice of anticoagulant in a patient with FX deficiency, the use of FXa inhibitor in our patient raised a handful of concerns and questions. Existing coagulation tests such as PT and aPTT levels do not accurately measure the therapeutic level of direct FXa inhibitor. Hence, the effectiveness of the direct FXa inhibitor used in patient with FX deficiency remained controversial. Studies have shown that chromogenic anti‐FXa assay may be useful when monitoring coagulation activity of direct FXa inhibitor. However, this test is not yet widely available.^10^, ^11^ This test was not performed in our patient as it was not available in our practice. The safety of apixaban used in our patient remained unclear as the clinical trials did not include patients with any form of severe bleeding disorder. In view of the above concerns, it is perhaps more justifiable to use apixaban at a lower recommended dose of 2.5 mg twice daily.

Several alternative treatment options exist for patients with concurrent AF and acquired FX deficiency such as dabigatran (a direct thrombin inhibitor), warfarin, splenectomy, left atrial appendage (LAA) occlusion, or no anticoagulation with continuous rhythm monitoring with an insertable cardiac monitor (ICM). Dabigatran might be the drug of choice in our patient as the drug directly inhibited thrombin (factor II). The drug level could potentially be monitored closely with clotting ecarin assays and dilute thrombin time and chromogenic.^12^ Those tests could be used to accurately identify the therapeutic drug levels.^12^ However, these tests are still not widely available in the market. An antidote (idarucizumab) has also been approved by FDA for its use in case of a severe bleeding event.^13^

Warfarin could also potentially be used in our patient with frequent monitoring of the INR level. A higher INR therapeutic range is likely needed as INR of 2‐3 might underestimate the antithrombotic effect in a patient with FX deficiency and AF.^14^ Spleen was thought to be the major adsorption site for FX. There have been several case reports of successful treatment of FX deficiency via splenectomy.^15^ Hence, another possible treatment option would be splenectomy if we are considering long‐term anticoagulation for our patient. LAA occlusion is a seemingly promising treatment to reduce AF‐related strokes.^16^ The European Society of Cardiology issued a class IIb recommendation to LAA occlusion in patient with contraindication for long‐term anticoagulation, as in our patient who has acquired FX deficiency.^7^ This is a potential alternative treatment of choice for stroke prevention in such rare clinical entity.

As for our patient, apixaban 5 mg twice daily was started and continued based on the following points. First, data from the Global Registry on Long‐Term Oral Antithrombotic Treatment in Patients with Atrial Fibrillation (GLORIA‐AF) phase 2 has demonstrated that there is a paradigm shift on the use of anticoagulants across North America and Europe in patients with AF.^17^ Second, there is no absolute contraindication to use novel oral anticoagulants (NOACs) in patients with any form of bleeding disorder. Third, from the technical standpoint, oral anticoagulant is deemed easier to take as it is readily available in a pill form; in contrast, low‐molecular‐weight heparin (LMWH) is administered subcutaneously and long‐term use of it leads to injection hematoma and patient discomfort. Fourth, the long‐term use of LMWH as an effective anticoagulant therapy in AF has not been well established. Fifth, as shown in several meta‐analyses, the NOACs (apixaban, edoxaban, rivaroxaban, and dabigatran) are potentially superior and at the very least noninferior to warfarin in preventing stroke and systemic embolism in patients with nonvalvular AF.^18^, ^19^, ^20^, ^21^ Sixth, although the NOACs appear to have similar efficacy for stroke prevention in nonvalvular AF, apixaban is noted to have a better safety profile with a lower risk of major bleeding compared with the other NOACs and warfarin.^18^, ^19^, ^20^, ^21^, ^22^ It is also worth to take note that rivaroxaban is associated with a higher risk of major bleeding compared with the rest of the NOACs.^19^, ^20^

A combination of FX deficiency and AF is a rarely reported clinical entity. To our knowledge, this is the first reported case of a patient, with acquired FX deficiency and AF, treated with apixaban for the past one and a half years without any significant bleeding or thromboembolic event. In view of her unpredictable bleeding risk, we utilized the strategy of ICM‐guided anticoagulation. Patient would take novel oral anticoagulant only around the time of AF episode.^1^, ^9^ As this is a single case report, the general applicability and safety on the use of direct FXa inhibitor in patients with FX deficiency remains unclear. The utility of ICM‐guided anticoagulation remains unclear as no randomized trials have been conducted to demonstrate its efficacy for stroke prevention in long term.

## 5 CONCLUSION

Our report is intended to share our clinical experience in managing patients with AF and FX deficiency. Having a better understanding of the coagulation cascade would lead to a better selection of anticoagulation. This case also highlights the importance of weighing the risk of bleeding and thromboembolic event. If FXa inhibitors were used in such rare clinical entity, physician should ensure that there is a reliable way of monitoring the coagulation activity of the drug prior to committing the patient to long‐term anticoagulation.

## CONFLICT OF INTEREST

None declared.

## AUTHORSHIP

JLT: drafted and revised the manuscript. DF: provided the histopathological images and description of the images. KPL and AG: critically reviewed the manuscript.
